# Electrocardiographic Pathological Findings Caused by the SARS-CoV-2 Virus Infection: Evidence from a Retrospective Multicenter International Cohort Longitudinal Pilot Study of 548 Subjects

**DOI:** 10.3390/jcdd10020058

**Published:** 2023-01-31

**Authors:** Nicola Susca, Antonio Giovanni Solimando, Paola Borrelli, Donatello Marziliano, Francesco Monitillo, Pasquale Raimondo, Domenico Vestito, Agostino Lopizzo, Gaetano Brindicci, Mohammad Abumayyaleh, Ibrahim El-Battrawy, Annalisa Saracino, Salvatore Grasso, Natale Daniele Brunetti, Vito Racanelli, Francesco Santoro

**Affiliations:** 1 School of Medicine: Interdisciplinary of Medicine, Aldo Moro University of Bari, 70124 Bari, Italy; 2 Guido Baccelli Unit of Internal Medicine, Department of Precision and Regenerative Medicine and Ionian Area-(DiMePRe-J), School of Medicine, Aldo Moro University of Bari, 70124 Bari, Italy; 3 Laboratory of Biostatistics, Department of Medical, Oral and Biotechnological Sciences, G. d’Annunzio University of Chieti-Pescara, 66100 Chieti, Italy; 4 Internal Medicine Residency Program, School of Medicine, Aldo Moro University of Bari, 70124 Bari, Italy; 5 University Cardiology Unit, University Hospital Polyclinic of Bari, 70124 Bari, Italy; 6 Section of Anesthesia and Intensive Care, Department of Emergency and Organ Transplantation, Aldo Moro University of Bari, 70124 Bari, Italy; 7 Cardiology Unit, University Hospital Polyclinic of Bari, 70124 Bari, Italy; 8 Department of Cardiology, San Carlo Hospital, 85100 Potenza, Italy; 9 Unit of Infectious Diseases, University Hospital Polyclinic of Bari, 70124 Bari, Italy; 10 Department of Cardiology, University Hospital Mannheim, 68167 Mannheim, Germany; 11 Department of Cardiology and Angiology, Bergmannsheil University Hospitals, Ruhr University of Bochum, 44789 Bochum, Germany; 12 Department of Medical and Surgical Sciences, University of Foggia, 71122 Foggia, Italy

**Keywords:** cardiac impairment, COVID-19, electrocardiography, mortality

## Abstract

COVID-19 has threatened the capability of receiving and allocating patients in emergency departments (EDs) all over the world. This is a retrospective cohort study to explore the role of a simple procedure like an ECG to screen for the severity of COVID-19 on admission to the ED. For this study, 548 consecutive patients were enrolled in a multicenter international registry and stratified upon ECG on admission with a simple distinction between normal vs. abnormal rhythm. Among patients in the abnormal ECG group were those with heart rates higher than 100 beats per minute and/or atrial fibrillation. Survival in patients with normal ECG rhythm was deemed below 75% after 58 days and then stabilized, while survival in patients with abnormal ECG rhythm was deemed below 75% after 11 days and below 50% after 21 days. A multivariate analysis including abnormal rhythm, gender, age, diabetes, obesity, respiratory failure during hospitalization, heart failure during hospitalization, and abnormal rhythm was an independent predictor of death (HR 7.20 95% CI 3.63–14.28, *p* < 0.01). This finding, if confirmed in large prospective studies, is promising for identifying a cheap and simple procedure for patients in need of a closer look.

## 1. Introduction

SARS-CoV-2 can determine infections with very different clinical characteristics and severity, ranging from asymptomatic to acute respiratory distress syndrome (ARDS). In addition to the lung, the organ most affected by the virus, many other organs are adversely affected due to the widespread expression of its target receptors ACE and TMPRSS2 [1]. Emerging evidence suggests an important cardiac involvement, with consequences on patient outcome [2,3].

Among hospitalized patients with COVID-19, a variable percentage of patients, about 20–30%, can have electrophysiological disturbances [4]. Considering the mechanisms underlying the arrhythmias in COVID-19, both direct and indirect effects of SARS-CoV-2 infection can be recognized. In the first group, we can recognize direct cardiotoxicity, dysregulation of the RAAS, endothelial damage and thromboinflammation, immune-dysregulation-induced cytokine storm, and demand-supply mismatch [5], with the pivotal potential mechanisms being hypoxia, myocarditis, abnormal host immune response, myocardial ischemia, myocardial strain, electrolyte derangements, metabolic and endocrine implications [6], intravascular volume imbalances and drug side effects [5]. Dysautonomia is a common feature as well, especially in the form of postural orthostatic tachycardia syndrome (POTS) [7]. The various proposed mechanisms proposed to be responsible for POTS differ between the acute setting and the chronic one. First, the viral infection leads to a hyper-responsiveness of the immune system, with autoimmune inflammation and immunosuppression. Next, in the chronic phase, both immune and non-immune mechanisms can interact in driving the POTS. On top of that, hypovolemia, hydration state, and other factors co-intervene [7].

In this study, we aimed to: (1) Evaluate whether patients with SARS-CoV-2 infection present with a different rate of ECG abnormalities, suggesting cardiac involvement and (2) assess the prognostic implications of ECG in patients hospitalized because of COVID-19, as well as construct a multivariable model based on these findings. We, therefore, assessed whether the ECG anomalies could be linked to the action of the virus and whether this affected the patients’ mortality.

## 2. Materials and Methods

A total of 548 consecutive patients admitted with a COVID-19 diagnosis were enrolled in this retrospective longitudinal cohort study. Patients were enrolled from March 2020 to December 2020 from 4 European hospitals: the infectious diseases unit and intensive care unit of Hospital-University Polyclinic of Bari, Italy; the Department of Infectious disease, Vittorio Emanuele II Hospital, Bisceglie, Italy; the Department of Infectious Disease, San Carlo Hospital, Potenza, Italy; and the First Department of Medicine, Faculty of Medicine, University Medical Centre Mannheim, Germany. An ECG was performed for each at admission, and several anamnestic, biochemical, and other parameters were collected. SARS-CoV-2 testing was based on the protocol released by the local and Italian authorities and as previously described [8]. In detail, laboratory confirmation of SARS-CoV-2 was defined by a positive result on a real-time reverse transcriptase-polymerase chain reaction (RT-PCR) assay performed on nasopharyngeal swabs or lower respiratory tract aspirates. Chest X-ray and, when needed, thoracic computerized tomography (CT) scan were performed to confirm the diagnosis. Patients were further divided into (1) COVID-19 and normal rhythm detected by ECG group (sinus rhythm with heart rate between 60–100 beats per minute) and (2) COVID-19 and abnormal ECG, including all subjects with any rhythm abnormality detected by ECG testing. Among patients within the abnormal ECG group were included those with heart rates higher than 100 beats per minute and/or atrial fibrillation.

This study was approved by the institutional ethics board, which waived the need for informed consent, and was performed in accordance with the ethical standards laid down in the 1964 Declaration of Helsinki and its later amendments. The study considered all adults (aged ≥ 18 years) with laboratory-confirmed SARS-CoV-2 infection and admitted them as in-patients in both intensive and non-intensive care units.

Clinical data were collected from electronic health records, including age, sex, smoking habit, triage vital signs and presenting symptoms, comorbidities, current medications, laboratory test results (pre-defined disease-specific panel), and duration and outcome of follow-up.

### 2.1. ECG Analysis and Definitions

Twelve-lead standard ECGs were recorded on admission with a CARDIOLINE^®^ HD+ ECG machine. The ECGs were retrieved by our dedicated institutional ECG storage server and independently analyzed by two cardiologists (FS, FM). The ECGs were analyzed before proceeding with an assessment of clinical outcome, which was therefore unknown to the ECG readers.

### 2.2. Statistical Analysis

Descriptive analysis was carried out using mean and standard deviation or median and interquartile range (IQR) for the quantitative variables and percentages values for the qualitative ones. Normality distribution for quantitative variables was assessed by the Shapiro–Wilk test. Univariate comparisons were investigated between groups (ECG with normal and abnormal rhythm) and explicative variables using the Pearson chi-square test or the Fisher’s exact test for categorical data, the Student’s *t*-test for independent data, or non-parametric Wilcoxon rank-sum test when appropriate for continuous data. Survival analysis was performed by applying the Kaplan–Meier estimator and log-rank test for equality of survivor functions. The association with clinical features was analyzed with the Cox model of proportional hazards (hazard ratio (HR) and 95% CI), and the applicability assumption was evaluated by the Schoenfeld test. Statistical significance was taken at the <0.05 level. All analyses were performed using STATA software v15.1 (StataCorp, College Station, TX, USA).

## 3. Results

### 3.1. Descriptive Characteristics and Comparisons

Five hundred forty-eight adult patients were included. There were 327 (59.7%) females and 221 (40.7%) males, with a mean age of 61.8 ± 16.9 years (range 18–98). Table 1 shows clinical, laboratory, and socio-demographic characteristics for all patients and stratified by ECG status (normal and abnormal rhythm). We found statistically significant differences between ECG status and outcome (χ^2^ = 52.66, df = 1, *p* < 0.001) and heart failure during hospitalization (χ^2^ = 13.24, df = 1, *p* < 0.001).

### 3.2. Survival Analysis

Survival in patients with normal ECG rhythm was deemed below 75% after 58 days and then stabilized, while survival in patients with abnormal ECG rhythm was deemed below 75% after 11 days and below 50% after 21 (Table 2).

**Figure 1:**
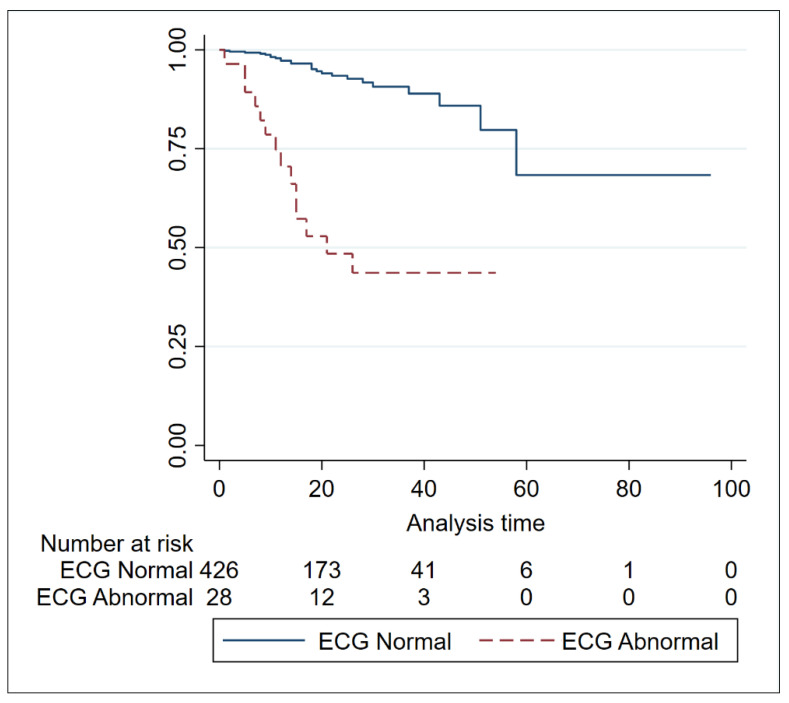
Kaplan–Meier curve of survival freedom among patients with normal and abnormal ECG.

At any time, median survival was always higher in subjects with a normal ECG rhythm than in those with an abnormal rhythm. This result was deemed statistically significant in both uni- and multivariate analyses (Figure 1 and Table 3).

Hazard ratio and corresponding 95% CIs were determined in univariate analyses through the Cox model for overall survival to evaluate relationships between ECG tracings and overall survival, showing a statistically significant HR both at univariate (HR = 8.92, 95%CI 4.60–17.26, *p* < 0.001) and multivariate analysis (HR = 7.20, 95%CI 3.63–14.28, *p* < 0.001).

Furthermore, a higher death rate was observed in patients affected by heart failure (HR = 3.98, 95%CI 2.10–7.53, *p* < 0.001) in univariate and multivariate analyses (HR = 2.74, 95%CI 1.33–5.72, *p* < 0.001). No significant association was appreciated between sex, diabetes, obesity and lung failure, and overall survival.

## 4. Discussion

A major pathological step of SARS-CoV-2 is the trigger function exerted in developing cardiac arrhythmias. Despite the caveats existing in the given rhythm disturbance, Guo et al. defined malignant rhythm disturbance as sustained ventricular tachycardia persisting for more than 30 s, with hemodynamic instability or ventricular fibrillation [9]. The authors uncovered troponin as a potential prognostic discriminator associated with rhythm disturbances in this scenario. The underlying mechanisms of arrhythmias in COVID-19 can be comprised within the clinical consequence of the acute myocardial injury, electrical instability associated with QT elongation, e.g., associated with hypokalemia, hypomagnesemia, and drug use. In addition, a key role is also played by the direct electrophysiological effect of cytokines on the myocardium, with a prolongation in the duration of the action potential and consequent demodulation of the calcium and potassium [10] channels. There is often a cytokine hyperactivation of the sympathetic system centrally and peripherally, as well as electrolyte abnormalities resulting from an alteration of renal function [11]. Despite these data, shreds of evidence regarding rapid and effective point-of-care ECG monitoring potentially stratifying COVID-19 based on simpler rhythm classification are scanty. While acknowledging this study’s limitations due to the lack of statistical power and the need for prospective trials to validate our hypothesis-generating report, to our knowledge, this is the first report highlighting the potential role of a basic ECG-oriented screening of patients at risk of a worse prognosis. This observation holds the potential to implement the already validated prognosticators’ tools [8,12] with a potential impact on outpatient settings besides hospital-admitted subjects, also in the era of effective outpatient therapies [13,14,15].

The value of this study lies in demonstrating the strong association with an unfavorable outcome of a simple and easily achievable parameter such as heart rhythm analysis.

A fair number of studies have deepened the prognostic role of ECG in COVID-19 patients, finding significant combinations of various parameters with increased mortality or critical illness: signs of previous myocardial infarction [16], acute change in the ST tract and T wave [16,17], left bundle branch block [18], intraventricular block [19], premature atrial beats [19], right bundle branch block [19], right ventricular strain [20], fragmented QRS [21], heart rate variability [22], poor R wave progression [23], and lengthening of QTc interval and subsequent development of life-threatening arrhythmias [3]. COVID-19 increases the risk of myocarditis, but it appears that direct myocardial involvement in SARS-CoV-2 infection is relatively rare [24] compared to extensive evidence of ECG alterations or increased heart damage enzymes. Similarly, SARS-CoV-2 increases the risk of acute myocardial infarction [25], but a presentation with a classic ST tract elevation remains rare [19]. In summary, heart damage is strongly associated with COVID-19 mortality [26]. With this work, we propose the simple evaluation of the rhythm at ECG (altered rhythm vs. normal rhythm) as an important piece for the prognostic stratification of the patient at admission to the emergency department, potentially being considered as a standing alone factor associated with increased mortality. After adequate validation as part of prospective studies, heart rhythm assessment could be used, along with other simple clinical, instrumental, and laboratory evaluations (such as the presence of dyspnea, chest ultrasound, and arterial blood gas analysis) as a screening tool in territorial structures and in a non-hospital and outpatient setting to direct the patient to a higher intensity treatment path. Moreover, based on resourceful in silico predictors available [27], a novel therapeutical landscape can be better tailored based on the given cardiological profiling.

## 5. Conclusions

Collectively, even though our aim is far from considering this study as a turning point in COVID-19 early management, its significance can be acknowledged as it could integrate and improve already existing prognostic indicators by recognizing pathological cardiac rhythm. An early and simple assessment of patients can be instrumental in providing the most appropriate and accurate allocation, especially in the outpatient setting, namely, in general practice.

## Figures and Tables

**Table 1:** Patient characteristics and comparison between patients with ECG normal and ECG abnormal (*n* = 548).

		ECG		
	Total	Normal	Abnormal	*p* Value
	N = 548	N = 510	N = 38	
Outcome				
Alive	508 (92.7%)	484 (94.9%)	24 (63.2%)	<0.001 ***
Dead	40 (7.3%)	26 (5.1%)	14 (36.8%)	
Gender				
Male	221 (40.3%)	205 (40.2%)	16 (42.1%)	0.817 **
Female	327 (59.7%)	305 (59.8%)	22 (57.9%)	
Hypertension				
Absent	459 (83.8%)	430 (84.3%)	29 (76.3%)	0.197 **
Present	89 (16.2%)	80 (15.7%)	9 (23.7%)	
Obesity				
Absent	476 (86.9%)	445 (87.3%)	31 (81.6%)	0.318 **
Present	72 (13.1%)	65 (12.7%)	7 (18.4%)	
Respiratory failure during hospitalization				
Absent	460 (83.9%)	432 (84.7%)	28 (73.7%)	0.074 **
Present	88 (16.1%)	78 (15.3%)	10 (26.3%)	
Heart failure during hospitalization				
Absent	441 (80.5%)	419 (82.2%)	22 (57.9%)	<0.001 **
Present	107 (19.5%)	91 (17.8%)	16 (42.1%)	
Anemia				
Absent	480 (94.1%)	445 (93.9%)	35 (97.2%)	0.412 ***
Present	30 (5.9%)	29 (6.1%)	1 (2.8%)	
Anticoagulant therapy				
Absent	351 (73.6%)	328 (74.2%)	23 (65.7%)	0.273 **
Present	126 (26.4%)	114 (25.8%)	12 (34.3%)	
Anticoagulant profilaxis				
Absent	147 (33.6%)	134 (33.1%)	13 (39.4%)	0.461 **
Present	291 (66.4%)	271 (66.9%)	20 (60.6%)	
Age (years)	61.8 ± 16.9	61.5 ± 16.7	66.2 ± 18.1	0.099 *

N (%) or mean and standard deviation or median (IQR) are shown when appropriate; * Student’s *t*-test for independent data ** Pearson’s chi-square test or *** Fisher’s exact test.

**Table 2:** ECG rhythm and survival.

				Survival Time		
ECG Rhythm	Time at Risk	Incidence Rate	N. of Subjects	25%	50%	75%
Normal	8605	0.0029053	426	58	NA	NA
Abnormal	566	0.024735	28	11	21	NA
Total	9171	0.0042525	454	58	NA	NA

**Table 3:** Cox model for overall survival. Univariate and multivariate correlation between abnormal ECG tracing, heart failure, and overall survival (OS).

	Cox Model for Overall Survival			
	Univariate Analysis		Multivariate Analysis	
	HR (95%CI)	* p * Value	HR (95%CI)	* p * Value
ECG (Ab vs. N)	8.92 (4.60−17.26)	<0.001	7.20 (3.63−14.28)	<0.001
Sex (M vs. F)	0.74 (0.39−1.39)	0.357	0.67 (0.33−1.33)	0.255
Age	1.03 (1.01−1.05)	0.003	1.00 (0.98−1.03)	0.518
Diabetes (Yes vs. No)	1.48 (0.72−3.02)	0.277	-	-
Obesity (Yes vs. No)	1.61 (0.73−3.52)	0.230	-	-
Respiratory failure during hospitalization (Yes vs. No)	2.44 (1.27−4.72)	0.007	1.25 (0.60−2.59)	0.534
Heart failure during hospitalization (Yes vs. No)	3.98 (2.10−7.53)	<0.001	2.74 (1.33−5.72)	0.006

## Data Availability

The data presented in this study are available on request from the corresponding author.
